# Intratumoral expression of interleukin 23 variants using oncolytic vaccinia virus elicit potent antitumor effects on multiple tumor models via tumor microenvironment modulation

**DOI:** 10.7150/thno.56494

**Published:** 2021-05-03

**Authors:** Lingjuan Chen, Hongqi Chen, Junjie Ye, Yan Ge, Haiyan Wang, Enyong Dai, Jinghua Ren, Weilin Liu, Congrong Ma, Songguang Ju, Z. Sheng Guo, Zuqiang Liu, David L. Bartlett

**Affiliations:** 1 Allegheny Health Network Cancer Institute, Pittsburgh, PA 15212, USA.; 2 Department of Surgery, Drexel University College of Medicine, Philadelphia, PA 19104, USA.; 3 UPMC Hillman Cancer Center and Department of Surgery, University of Pittsburgh School of Medicine, Pittsburgh, PA 15213, USA.; 4 Cancer Center, Union Hospital, Huazhong University of Science and Technology, Wuhan, Hubei Province, China.; 5 Shanghai Jiao Tong University Affiliated Sixth People's Hospital, Shanghai, China.; 6 Department of Cancer Center, Renmin Hospital of Wuhan University, Wuhan, Hubei Province, China.; 7 Department of Immunology, School of Biology and Basic Medical Sciences, Medical College, Soochow University, Suzhou, Jiangsu Province, China.; 8 Department of Oncology and Hematology, China-Japan Union Hospital of Jilin University, Changchun, Jilin Province, China.; 9 Xiangya School of Medicine, Central South University, Changsha, Hunan Province, China.

**Keywords:** oncolytic virus, IL-23, cancer immunotherapy, tumor microenvironment, efficacy.

## Abstract

**Background:** Newly emerging cancer immunotherapy has led to significant progress in cancer treatment; however, its efficacy is limited in solid tumors since the majority of them are “cold” tumors. Oncolytic viruses, especially when properly armed, can directly target tumor cells and indirectly modulate the tumor microenvironment (TME), resulting in “hot” tumors. These viruses can be applied as a cancer immunotherapy approach either alone or in combination with other cancer immunotherapies. Cytokines are good candidates to arm oncolytic viruses. IL-23, an IL-12 cytokine family member, plays many roles in cancer immunity. Here, we used oncolytic vaccinia viruses to deliver IL-23 variants into the tumor bed and explored their activity in cancer treatment on multiple tumor models.

**Methods:** Oncolytic vaccinia viruses expressing IL-23 variants were generated by homologue recombination. The characteristics of these viruses were *in vitro* evaluated by RT-qPCR, ELISA, flow cytometry and cytotoxicity assay. The antitumor effects of these viruses were evaluated on multiple tumor models *in vivo* and the mechanisms were investigated by RT-qPCR and flow cytometry.

**Results:** IL-23 prolonged viral persistence, probably mediated by up-regulated IL-10. The sustainable IL-23 expression and viral oncolysis elevated the expression of Th1 chemokines and antitumor factors such as IFN-γ, TNF-α, Perforin, IL-2, Granzyme B and activated T cells in the TME, transforming the TME to be more conducive to antitumor immunity. This leads to a systemic antitumor effect which is dependent on CD8^+^ and CD4^+^ T cells and IFN-γ. Oncolytic vaccinia viruses could not deliver stable IL-23A to the tumor, attributed to the elevated tristetraprolin which can destabilize the IL-23A mRNA after the viral treatment; whereas vaccinia viruses could deliver membrane-bound IL-23 to elicit a potent antitumor effect which might avoid the possible toxicity normally associated with systemic cytokine exposure.

**Conclusion:** Either secreted or membrane-bound IL-23-armed vaccinia virus can induce potent antitumor effects and IL-23 is a candidate cytokine to arm oncolytic viruses for cancer immunotherapy.

## Introduction

Conventional cancer therapies generally target the proliferation, survival, or metabolic activity of tumor cells directly 1, whereas newly emerging cancer immunotherapies restore anticancer immunity by modulating the tumor microenvironment (TME), tipping the equilibrium between factors that stimulate or inhibit anticancer immunity - tipping the cancer-immune set point 2. Modern cancer immunotherapies, including immune checkpoint blockade, adoptive cell transfer, and cancer vaccines are ultimately dependent on immune cells (especially T cells) for their antitumor effects. However, the majority of solid tumors are characterized by a paucity of intratumoral T cell infiltrate, and defined as non-T cell-inflamed or “cold” tumors 3, 4. Therefore, new approaches that can improve intratumoral T cell infiltrate and transform “cold” tumors into “hot” or T cell-inflamed tumors are urgently needed to improve the efficacy of cancer immunotherapy.

Oncolytic viruses can directly kill tumor cells like conventional cancer therapies, and this killing is tumor-selective owing to the oncolytic characteristic of these viruses. This killing further provides a natural repertoire of tumor-associated antigens (TAAs), danger signals (including damage-associated molecular pattern (DAMP) and OV-derived pathogen-associated molecular pattern (PAMP) molecules) and inflammatory cytokines to trigger innate and adaptive antitumor immunity. This antitumor immune reactivity results in the infiltration of diverse immune cells, including T lymphocytes, into the TME 5, 6. Oncolytic viruses have positive effects on almost every aspect of the cancer-immunity cycle and can be further armed with chemokines, cytokines, or other molecules to modulate the TME so as to harness the immune system to attack and treat tumors 7-11. Thus, oncolytic viral therapy falls into either of the two broad categories of immunotherapy approaches: direct approaches which primarily modify the tumor cells itself; indirect approaches predominately targeting the TME 12. In this sense, oncolytic viruses can be considered as a form of immunotherapy 13 and has been suggested to be the next remarkable wave in cancer immunotherapy 14.

Variable oncolytic viruses either armed or unarmed have been successfully applied in the transformation of “cold” tumors into “hot” tumors and elicited antitumor effects 15-27. A tumor-selective oncolytic vaccinia virus (vvDD) with thymidine kinase (TK) and vaccinia growth factor (VGF) deficiency is safe in clinical trials 28-30. Intratumoral expression of the chemokines CXCL11 20 and IL-15Rα 21 using vvDD have proven to be efficient in the transformation of solid tumors and the induction of antitumor effects. In order to avoid cytokine release syndrome, membrane-bound IL-2 or IL-12 have also been expressed by tumor cells using vvDD. These viruses not only diminished toxic side effects associated with systemic exposure, but also transformed the TME and treated several murine tumor models, especially in combination with PD-1 blockade, curing all or most of the mice with a high tumor burden 27, 31.

IL-23 is another cytokine in the IL-12 cytokine family. IL-12 is formed by the pairing of IL-12p40 and IL-12p35 subunits, whereas IL-23 is formed by the pairing of IL-12p40 and IL-23p19 or IL-23A subunits. IL-12 receptor is composed of IL-12β1 and IL-12β2, whereas IL-23 receptor is composed of IL-12β1and IL-23R. Unlike IL-12 with well-recognized antitumor effects 32, IL-23 activity is more controversial. In general, endogenous IL-23 is suggested to promote tumor growth, demonstrated with many tumor models using IL-23A- or IL-23 receptor-deficient mice, while exogenous IL-23 is suggested to suppress tumor growth, demonstrated with many IL-23-overexpressing tumor cell lines 33. The antitumor effect of IL-23 was also demonstrated using viral delivery 34-36. However, a recent study also demonstrated the overexpression of IL-23A in murine breast cancer 4T1 cells promoted tumor growth via TME modulation 37. The goal of our current study was to investigate whether and how the overexpression of IL-23 variants using an oncolytic vaccinia virus modulates the TME and further improves antitumor effects using multiple tumor models.

## Materials and methods

### Mice and cell lines

Female C57BL/6 (B6 in short) and BalB/c mice were purchased from The Jackson Laboratory (Bar Harbor, ME) and housed in specific pathogen-free conditions in the University of Pittsburgh Animal Facility and Allegheny Health Network Research Institute Preclinical Facility. All animal studies were approved by the University of Pittsburgh Institutional Animal Care and Use Committee and Allegheny Health Network Research Institute Institutional Animal Care and Use Committee. Murine colon cancer MC38-luc, ovarian cancer ID-8-luc, and mesothelioma AB12-luc cells were generated by the infection of parental tumor cells with firefly luciferase-carrying lentivirus and antibiotic blasticidin selection. Normal African green monkey kidney fibroblast CV1, Human embryonic kidney 293 (HEK293) cells, murine melanoma B16, colon cancer CT26, breast cancer EMT6 and Lewis lung cancer (LLC) cells were obtained from American Type Culture Collection. HEK293 cells were grown in Dulbecco's Modified Eagle's medium (DMEM) supplemented with 20% Calf Bovine Serum (CBS), 2 mM L-glutamine, and 1 x penicillin/streptomycin in a 37 °C, 5% CO_2_ incubator. Other cell lines were grown in DMEM supplemented with 10% FBS, 2 mM L-glutamine, and 1 x penicillin/streptomycin in a 37 °C, 5% CO_2_ incubator.

### Virus generation

VSC20, a *vgf* gene-deleted Western Reserve strain vaccinia virus, was used as the parental virus for homologous recombination. IL-23A cDNA was amplified from pGEM-IL-23A (Sino Biological, Wayne, PA) using PCR (Forward primer: GGCGGTCGACATGCTGGATTGCAGAGCAGTAATA; Reverse primer: CGCGGGCGCGCCTTAAGCTGTTGGCACTAAGGGC). The cDNA fragment was digested with SalI+AscI, and ligated via T4 ligase into plasmids pCMS1-IRES-YFP, pCMS1-IRES-IL-12p40-YFP, or pCMS1-IRES-IL-12p40-FG-YFP 31, resulting in new shuttles plasmids pCMS1-IL-23A-YFP, pCMS1-IL-23A-IRES-IL-12p40-YFP or pCMS1-IL-IL-23A-IRES-IL-12p40-FG-YFP, respectively. All these shuttle vectors were used for homologous recombination of murine *IL-23* variants plus yellow fluorescence protein (YFP) marker into the *tk* locus of the vaccinia viral genome of VSC20. To make the new viruses vvDD-IL-23A, vvDD-IL-23, and vvDD-IL-23-FG, CV-1 cells were infected with VSC20 at a multiplicity of infection (MOI) of 0.1 and then transfected with the shuttle plasmids, resulting in virus mixture. Selection of the new recombinant viruses was based on expression of YFP in CV1 cells 24 h after the infection of relative virus mixture. A double viral gene-deficient (*tk-* and *vgf-*) vaccinia virus carrying *yfp* cDNA at its *tk* locus, vvDD-YFP (vvDD in short), was the control virus for this work.

### Viral replication and IL-23 expression *in vitro*

MC38-luc (3×10^5^), B16 (2×10^5^), or AB12-luc (3×10^5^) cells were seeded in 24-well plates overnight and infected with vvDD, vvDD-IL-23A, vvDD-IL-23, or vvDD-IL-23-FG at an MOI of 1 in 0.15 mL 2% FBS-containing-DMEM for 2 h and 0.35 mL 10% FBS-containing-DMEM was added to cells. After 24 h culture, the supernatants were harvested to measure IL-23 using enzyme-linked immunosorbent assay (ELISA) (BD Bioscience, San Jose, CA) and the cell pellets were applied either to measure membrane-bound IL-23 using flow cytometry or to extract RNA to measure the viral house-keeping gene A34R to monitor viral replication and transgene IL-23 expression by RT-qPCR, respectively. To further confirm IL-23 expression, the tumor cells were infected with indicated viruses at MOIs of 0.1, 1, and 5 and harvested 24 h post-infection to measure IL-23 using ELISA. In some experiment, tumor cells were infected with vvDD at an MOI of 1 to measure IL-23R expression 24 h after infection by flow cytometry.

### Cytotoxicity assay *in vitro*

Tumor cells were plated at 8×10^3^ (except MC38-luc cells, which were plated at 1.0×10^4^) cells per well in 96-well plates and infected with indicated viruses the next day at different MOIs. Cell viability was determined at 48 h after infection using Cell Counting Kit-8 (Boster Biological Technology, Pleasanton, CA).

### Rodent tumor models

B6 mice were intraperitoneally (i.p.) inoculated with 5×10^5^ MC38-luc cancer cells or 3.5×10^6^ ID8-luc cancer cells, respectively, and divided into required groups at the indicated day post-tumor cell inoculation according to tumor size based on live animal IVIS imaging, performed using a Xenogen IVIS 200 Optical *In Vivo* Imaging System or IVIS Lumina LT Series III (Caliper Life Sciences, Hopkinton, MA). Grouped mice were i.p. injected with indicated viruses, or PBS. In some experiments, anti-CD8 Ab (clone 53-6.7; Bio X Cell; 250 µg per injection), anti-CD4 Ab (clone GK1.5, Bio X Cell; 150µg per injection), or anti-IFN-γ Ab (clone XMG1.2, Bio X Cell; 200 µg per injection) were i.p. injected into mice to deplete CD8^+^ T cells, CD4^+^ T cells, or neutralize circulating IFN-γ, respectively. In some experiments, mice were sacrificed to harvest individual peritoneal tumor nodules for further analysis.

B6 mice were subcutaneously (s.c.) inoculated with 2×10^5^ B16, 5×10^5^ or 1×10^6^ LLC in the right flank or BalB/c mice were s.c. inoculated with 1×10^6^ CT26 in the right flank or 1×10^6^ EMT6 in the mammary fat pad. Tumor-bearing mice were treated with 60 µL PBS or 5×10^7^ PFU (plaque forming units) /60 µL virus per mouse via intratumorally (i.t.) injection at indicated days after tumor cell inoculation. MC38-luc-tumor-bearing B6 mice treated with vvDD-IL-23, which had survived for more than 120 days, were s.c. challenged with 1×10^6^ MC38. Naïve B6 mice also received the same dose tumor challenge as a control. Subcutaneous tumor size was measured using an electric caliper in two perpendicular diameters.

### Flow cytometry

Collected tumor tissues were weighed and incubated in RPMI 1640 medium containing 2% FBS, 1 mg per ml collagenase IV (Sigma: #C5138), 0.1 mg hyaluronidase (Sigma: #H6254), and 200U DNase I (Sigma: #D5025) at 37 °C for 1-2 h to make single cells. Single cells from tumor tissues or i*n vitro* virus-infected cells were blocked with α-CD16/32 Ab (clone 93, eBioscience: #14-0161-85; 1:1000) and then stained with antibodies against mouse CD45 (PerCP-Cy5.5 or FITC, clone: 30-F11, BioLegend: #103132 or 103108; 1:300), CD11b (PE, clone: M1/70, BioLegend, #101208; 1:300), Ly-6G (APC, clone: 1A8, BioLegend, #127614; 1:300), Ly-6C (FITC, clone: HK1.4, BioLegend, #128016; 1:300), F4/80 (FITC, clone: BM8, BioLegend, # 123116; 1:300), CD4 (APC, clone: RM4-5, eBioscience: #17-0042-81; 1:300), Foxp3 (PE, clone: FJK-16s, eBioscience: #12-5773-82; 1:100), CD8 (PE or APC, clone: 53-6.7, eBioscience: #12-0081-85 or 17-0081-83; 1:300), IFN-γ (PE, clone: XMG1.2, BioLegend, #505808; 1:100) and TNF-α (PE, clone: MP6-XT22, BioLegend, #506306; 1:100) and IL-12p40 (PE, clone: C17.8, eBioscience: #12-7123-82; 1:300). The intracellular staining kit for Foxp3, IFN-γ and TNF-α staining was purchased from BioLegend. Samples were collected on a BD Accuri C6 cytometer and data were analyzed using BD Accuri C6 cytometer software.

### RT-qPCR

Total RNA was extracted from viral-infected cells or tumor tissues using the RNeasy Kit (Qiagen, Valencia, CA). One microgram of RNA was used for cDNA synthesis, and 25 to 50 ng of subsequent cDNA was used to conduct mRNA expression TaqMan analysis on the StepOnePlus system (Life Technologies, Grand Island, NY). All primers for the analysis were purchased from Thermo Fisher Scientific (Waltham, MA). Gene expression was normalized to the housekeeping gene HPRT1 and expressed as fold increase (2^-ΔCT^), where ΔCT = CT_(Target gene)_ - CT_(HPRT1)_.

### Statistics

Statistical analyses were performed using unpaired Student's *t* test (GraphPad Prism version 9). Data are means ± SD. Animal survival is presented using Kaplan-Meier survival curves and was statistically analyzed using a log-rank test (GraphPad Prism version 9). Tumor growth cures were statistically analyzed using two-way ANOVA (GraphPad Prism version 9). Values of *P* < 0.05 were considered statistically significant, and all *P* values were two-sided. In the figures, standard symbols are used: * *P* < 0.05; ** *P* < 0.01; *** *P* < 0.001; and **** *P* < 0.0001.

### Data availability

All data are available from the corresponding author upon reasonable request.

## Results

### IL-23 expression does not impact viral replication and cytotoxicity *in vitro*

**Figure 1:**
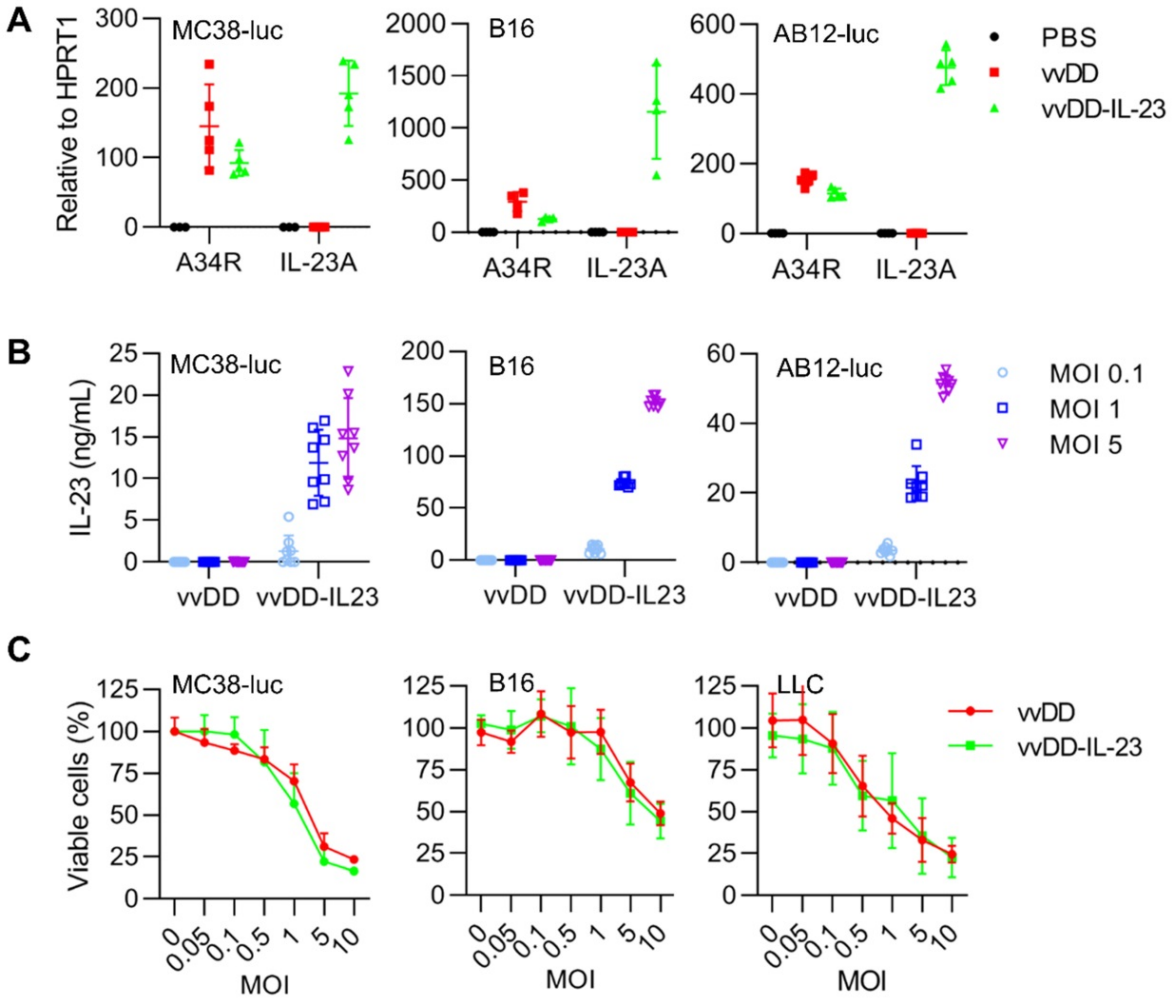
** vvDD-IL-23 infection shows significantly higher IL-23 secretion and similar replication and cytotoxicity *in vitro*.** (**A**) Tumor cell MC38-luc (3×10^5^ cells), B16 (2×10^5^ cells) or AB12-luc (3×10^5^ cells), were mock-infected or infected with vvDD or vvDD-IL-23 at an MOI of 1. The cell pellets were harvested to measure A34R or IL-23 expression 24 h after infection using RT-qPCR. (**B**) MC38-luc (3×10^5^ cells), B16 (2×10^5^ cells) or AB12-luc (3×10^5^ cells) were mock-infected or infected with vvDD or vvDD-IL-23 at MOIs of 0.1, 1, and 5. The supernatants were harvested to measure IL-23 using ELISA 24 h after infection. (**C**) MC38-luc (1×10^4^ cells), B16 (8×10^3^ cells) or LLC (8×10^3^ cells) were infected with vvDD-IL-23 or vvDD at indicated MOIs and cell viability was measured 48 h after infection.

To explore the function of IL-23 in cancer, we used vvDD, a double viral gene-deficient (*tk-* and *vgf-*) oncolytic vaccinia virus, to express murine IL-23 and this new virus was called vvDD-IL-23 (Figure S1). When MC38-luc, AB12-luc, and B16 cells were infected with vvDD-IL-23 or control virus (vvDD) at an MOI of 1, viral housekeeping gene (A34R) mRNA levels were similar between these two viruses, while IL-23 mRNA levels (measured by IL-23A) were significantly higher in vvDD-IL-23, as expected (Figure 1A). We further measured the IL-23 amount in cell culture supernatants after vvDD-IL-23 infection with MOIs 0.1, 1 or 5. The amount of IL-23 positively correlated with virus MOIs (Figure 1B). These data showed the recombinant virus was successfully constructed. To investigate the possible impact of foreign gene expression on virus cytotoxicity, we infected MC38-luc, B16 and LLC cells with vvDD-IL-23 or control virus vvDD at MOIs of 0.05, 0.1, 0.5, 1, 5 or 10, respectively, and measured the cell viability 48 h after the infection. The data suggested the IL-23 expression did not significantly impact the viral cytotoxic capacity (Figure 1C).

### IL-23 expressing oncolytic virus elicits antitumor effects in multiple tumor models

**Figure 2:**
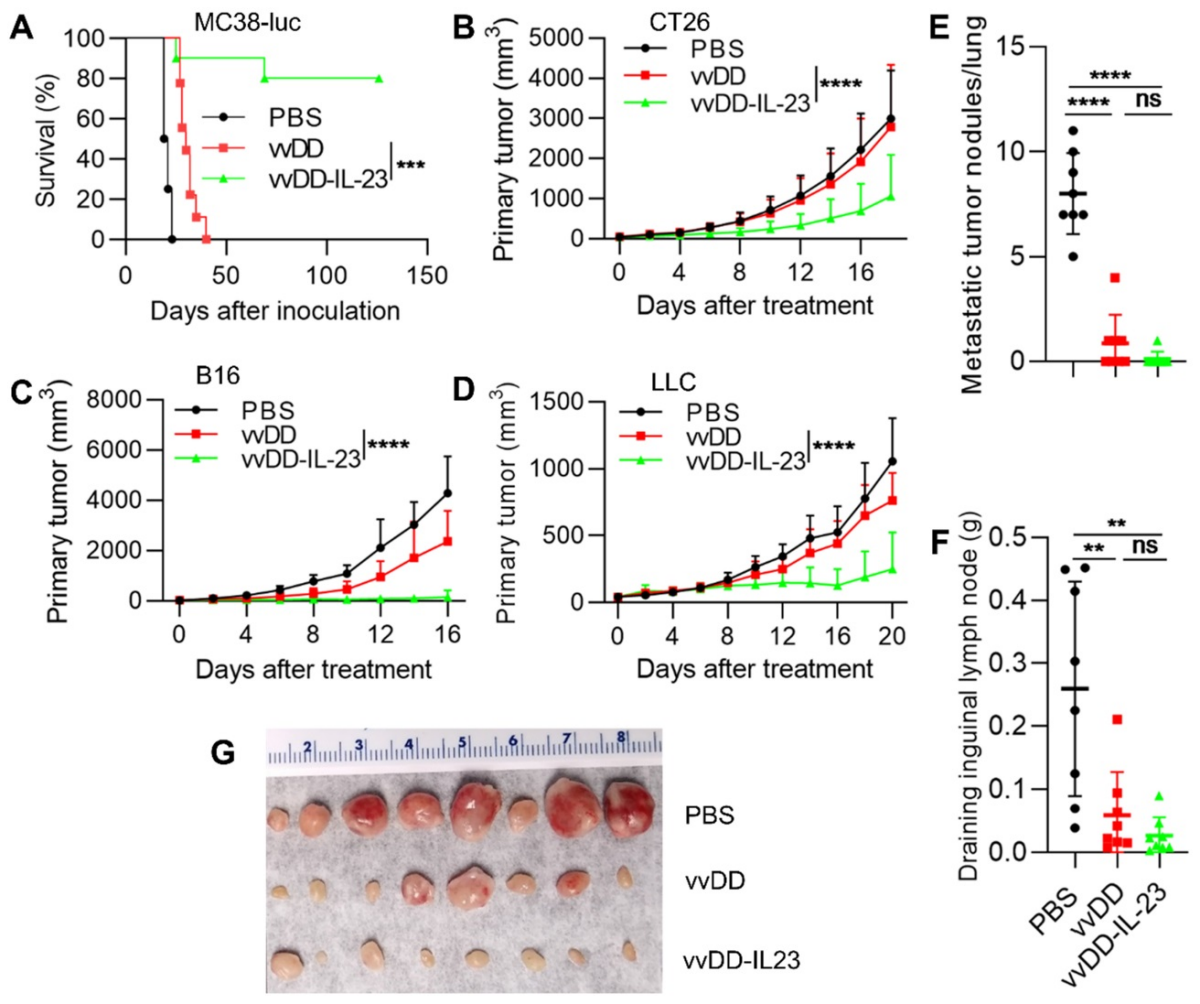
** vvDD-IL-23 treatment elicits potent therapeutic effects in multiple tumor models.** (**A**) B6 mice were i.p. inoculated with 5×10^5^ MC38-luc cells and treated with PBS, vvDD, or vvDD-IL-23 at 2×10^8^ PFU/mouse five days after tumor inoculation. The Kaplan Meier survival curve is shown. BalB/c mice were s.c. inoculated with 1×10^6^ CT26 or B6 mice were s.c. inoculated with 2×10^5^ B16 or 5×10^5^ LLC in the right flanks, and were i.t. treated with 60 µL PBS or 5×10^7^ PFU/60 µL virus per mouse at day 6, 10 or 7 after tumor cell inoculation, respectively. Tumor growth curves are shown in (**B**), (**C**) and (**D**), respectively. The endpoints were determined by natural death or tumor size over 2 cm. B6 mice were s.c. inoculated with 1×10^6^ LLC in the right flanks and sacrificed at the first mouse with a tumor size over 2 cm. The lung metastatic tumor nodules were counted (**E**). The individual draining inguinal lymph nodes were collected, weighed (**F**) and photographed (**G**). A log-rank (Mantel-Cox) test was used to compare survival rates. A two-way ANOVA test was used to compare tumor growth cures. **: *P*<0.01; ***: *P*<0.001; and **** *P*<0.0001. ns: not significant.

To evaluate the antitumor efficacy of vvDD-IL-23, we injected PBS, vvDD or vvDD-IL-23 at the dose of 2×10^8^ PFU per mouse i.p. to treat B6 mice bearing five-day old peritoneal murine colon cancer MC38-luc; survival results demonstrated that vvDD-IL-23 elicited significantly potent antitumor effects compared with PBS or vvDD treatment (Figure 2A). We also explored the therapeutic efficacy of vvDD-IL-23 using subcutaneous tumor models. BalB/c mice were s.c inoculated with tumor cells CT26 in the right flank or EMT6 in the mammary fat pad, and 6 days later the resulting tumor-bearing mice were i.t. injected with PBS, vvDD, or vvDD-IL-23. IL-23-expressing vvDD significantly retarded tumor growth or cured some mice compared with PBS and vvDD treatment. The CT26 tumor growth curve is shown in Figure 2B and the individual tumor growth pattern is shown in Figure S2A. The EMT6 tumor growth curve and individual tumor growth pattern are shown in Figure S2B-C. B6 mice were s.c. inoculated with tumor cells B16 or LLC in the right flank, and the resulting tumor-bearing mice were i.t. injected with PBS, vvDD, or vvDD-IL-23 at day 10 or day 7 after tumor cell inoculation, respectively. Tumor growth characteristics or individual tumor growth patterns were similar to the CT26 tumor model (Figure 2C-D; Figure S2B-E). We also evaluated the anti-metastatic effect of vvDD-IL-23 treatment using the LLC subcutaneous model. The results showed that both viral treatments significantly reduced the metastases both in lungs (Figure 2E) and draining inguinal lymph nodes (Figure 2F-G), and vvDD-IL-23 treatment worked slightly better in preventing metastases, though this difference was not significant when compared with vvDD treatment (Figure 2E-G). These data suggested a potent therapeutic effect was elicited by IL-23 expressing oncolytic vaccinia virus (vvDD-IL-23).

### IL-23 expressing oncolytic virus modulates the tumor microenvironment

**Figure 3:**
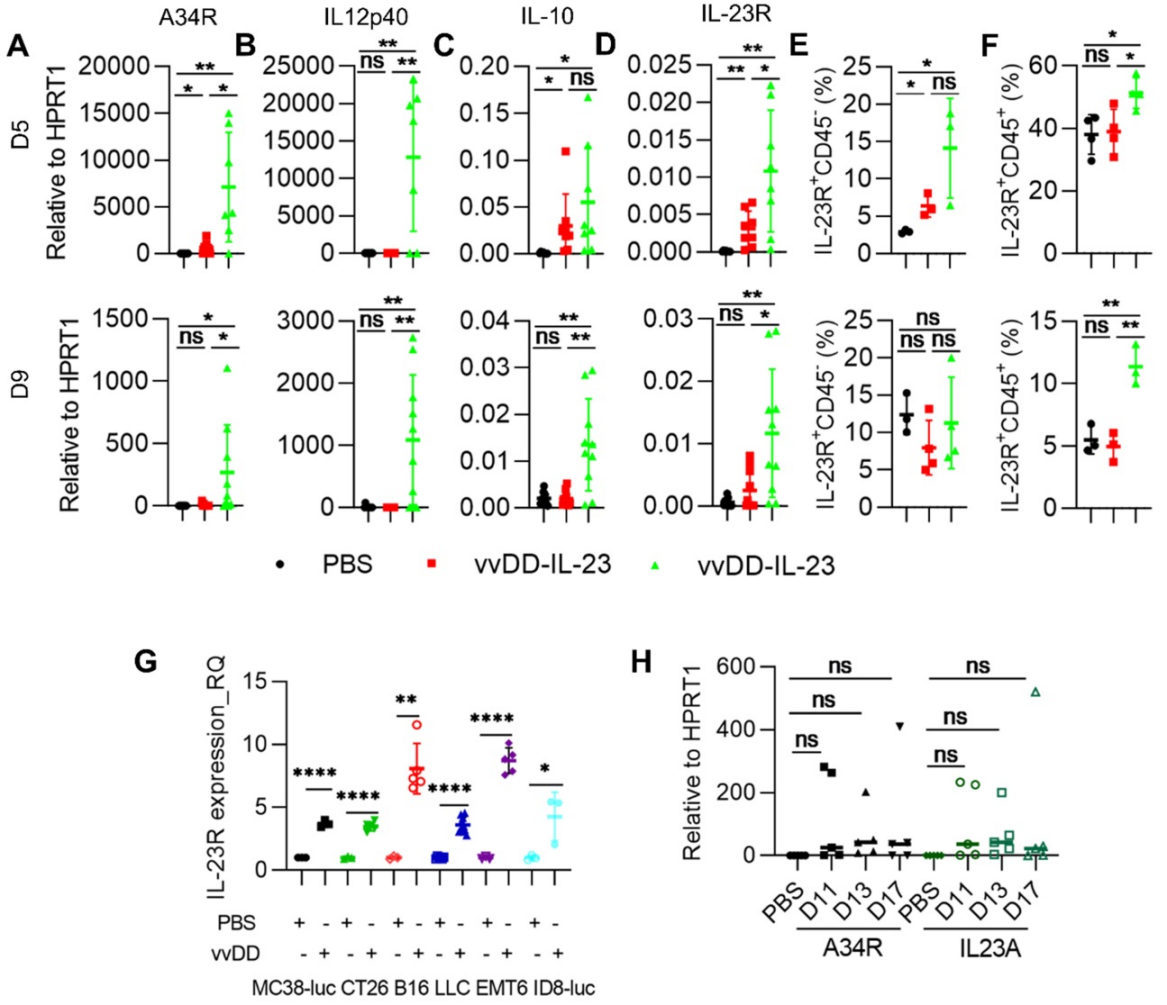
** vvDD-IL-23 treatment prolongs viral persistence.** B6 mice were i.p. inoculated with 5×10^5^ MC38-luc cells and treated with PBS, vvDD, or vvDD-IL-23 at 2×10^8^ PFU/mouse five days after tumor inoculation. Tumor-bearing mice were sacrificed five or nine days post-treatment and primary tumors were collected and analyzed using RT-qPCR to determine the expression of A34R (**A**), IL12p40 (**B**), IL-10 (**C**) and IL-23R (**D**). The IL-23R expression on CD45^-^ or CD45^+^ cells were determined by flow cytometry (**E**, **F**). MC38-luc (3×10^5^ cells), CT26 (3×10^5^ cells), B16 (2×10^5^ cells), LLC (3×10^5^ cells), EMT (3×10^5^ cells), or AB12-luc (3×10^5^ cells) tumor cells were mock-infected or infected with vvDD at an MOI of 1. The cell pellets were harvested to measure IL-23R expression at 24 h after infection using flow cytometry (**G**). MC38-luc-bearing mice treated as above were also sacrifice at D11, D13 and D17 to monitor viral persistence in tumors using RT-qPCR (**H**). *: *P*<0.05; **: *P*<0.01; and ****: *P*<0.0001. ns: not significant.

To explore the mechanisms by which vvDD-23 treatment elicits antitumor effects, we investigated the TME using the MC38-luc tumor model. B6 mice were i.p. inoculated with MC38-luc tumor cells and 5 days later i.p. injected with PBS, vvDD, or vvDD-IL-23. To investigate the dynamic modulation of the TME, treated mice were sacrificed at day 5 and day 9 after treatment and tumor nodules were collected for RT-qPCR analysis. We first measured the transgene expression. It is interesting to find that vvDD treatment significantly elevated the mRNA of the viral house-keeping gene A34R at day 5 but not day 9, compared with PBS treatment; whereas vvDD-IL-23 treatment significantly elevated the mRNA of the viral house-keeping gene A34R at both day 5 and day 9, compared with PBS or vvDD treatment (Figure 3A). Correspondingly, vvDD-IL-23 treatment also led to a significant increase of IL-12p40 at both day 5 and day 9 compared with PBS or vvDD treatment (Figure 3B). As this is in contrast to *in vitro* data which demonstrated similar viral infectivity and cytotoxicity between vvDD-IL-23 and vvDD, the *in vivo* results suggest that IL-23 expression might delay viral clearance from the tumor by the antiviral immune response. Since it was previously demonstrated that the immune suppressive cytokine IL-10 expressed by vaccinia virus prolonged viral persistence 38, and it is known that IL-23 can up-regulate IL-10 expression 39, we measured IL-10 mRNA levels in tumor nodules. At day 5, compared with PBS treatment, both virus treatments significantly elevated IL-10 mRNA, though this elevation was comparable between the two viruses. However, only vvDD-IL-23 treatment significantly elevated IL-10 mRNA levels in the tumor at day 9 (Figure 3C), suggesting that IL-10 might mediate the vvDD-IL-23 induced viral persistence in tumor. We also measured IL-23 receptor (IL-23R) in the tumor since IL-23R was reported to be elevated after IL-23 treatment 40, and found that its expression was positively correlated with IL-23 expression (Figure 3D). IL-23R expression on CD45^-^ cells was only increased on day 5, but IL-23R expression on CD45^+^ cells was increased both on day 5 and day 9 after vvDD-IL-23 treatment (Figure 3E-F), suggesting a role of IL-23/IL-23R positive feedback in the antitumor effect. The impact of prolonged viral replication on the antitumor immune response will need to be addressed in future studies. It is worth mentioning that vvDD treatment also significantly increased IL-23R expression in the tumor at day 5 after treatment (Figure 3D) and in the vvDD-infected cancer cells *in vitro* (Figure 3G), supporting a rational combination of vvDD and cytokine IL-23. We continued to monitor the viral persistence at D11, D13 and D17 after vvDD-IL-23 treatment. The results showed that the viruses in vvDD-IL-23-treated tumors were mostly cleared by D11, although a few of the tested mice had obvious virus persistence and IL-23 expression beyond D11 (Figure 3H).

**Figure 4:**
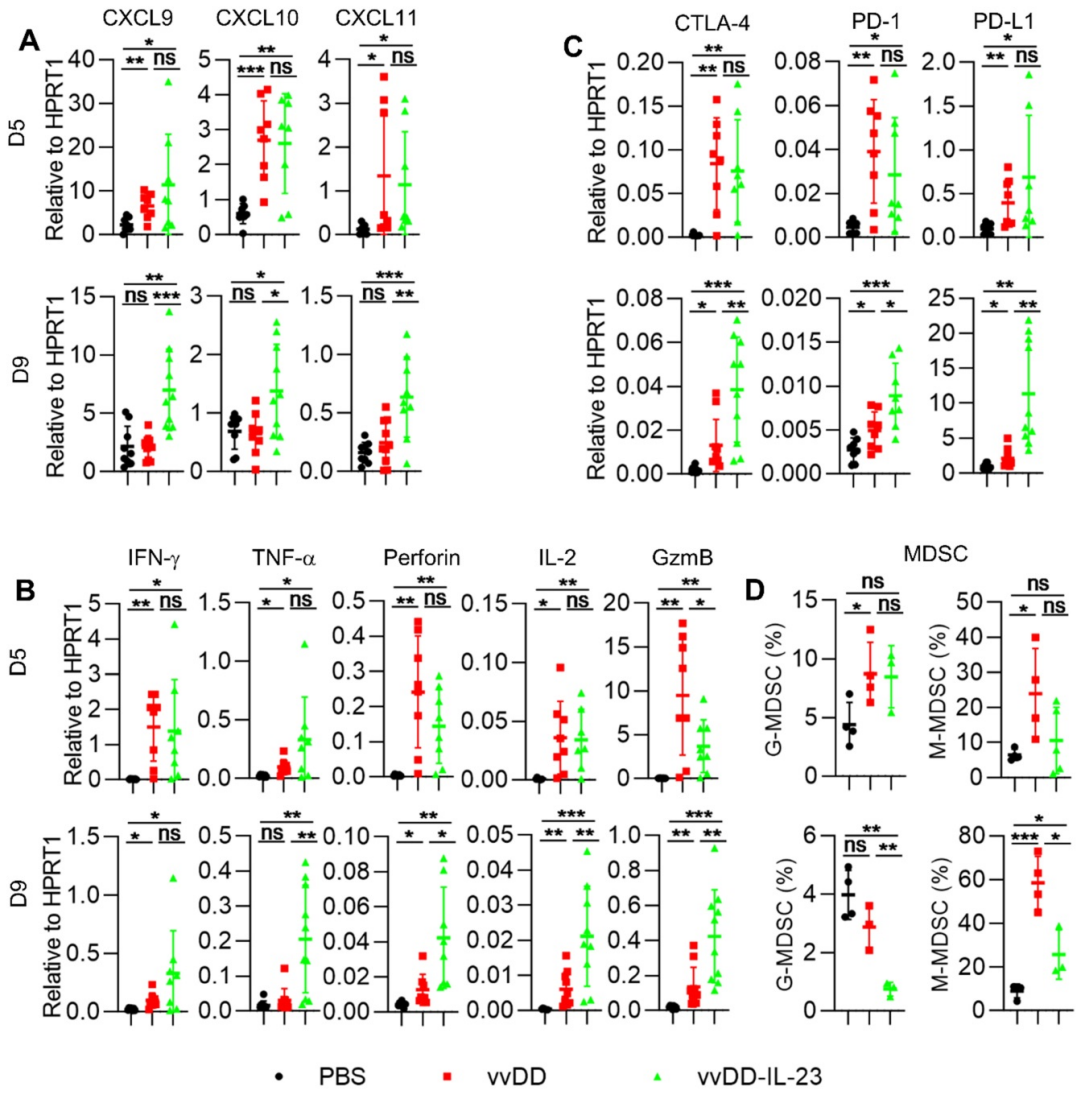
** vvDD-IL-23 treatment transforms TME.** B6 mice were i.p. inoculated with 5×10^5^ MC38-luc cells and treated with PBS, vvDD, or vvDD-IL-23 at 2×10^8^ PFU/mouse five days after tumor inoculation. Tumor-bearing mice were sacrificed five or nine days after treatment and primary tumors were collected and analyzed using RT-qPCR to determine the expression of Th1 chemokines (**A**), antitumor immunity mediators (**B**) and immune checkpoints (**C**) in the TME. The percentages of G-MDSC (CD45^+^CD11b^+^Ly-6G^+^Ly-6C^low^) and M-MDSC (CD45^+^CD11b^+^Ly-6G^-^Ly-6C^hi^) in tumor CD45^+^CD11b^+^ cells were determined by flow cytometry (**D**). *: *P*<0.05; **: *P*<0.01; and ***: *P*<0.001. ns: not significant.

We further measured the expression of Th1 chemokines and other mediators associated with anti-/pro- tumor immunity in the TME. At day 5 after treatment, both viral treatments induced significantly more Th1 chemokines (CXCL9, CXCL10 and CXCL11) compared with PBS treatment. However, only vvDD-IL-23 treatment induced significantly more Th1 chemokines at day 9 after treatment compared with either PBS or vvDD treatment (Figure 4A). Antitumor immunity associated factors IFN-γ and TNF-α had a similar expression pattern as Th1 cytokines (Figure 4B). Antitumor immunity associated factors perforin and IL-2 were elevated at both day 5 and day 9 after viral treatment, but at day 9, vvDD-IL-23 induced significantly more perforin and IL-2 than vvDD (Figure 4B). Both viral treatments induced significantly more of the antitumor factor granzyme B (GzmB) expression compared with PBS treatment at day 5 and day 9. However, the expression of GzmB was higher in vvDD-treated tumors than that in vvDD-IL-23-treated tumors at day 5 after treatment, whereas was lower in vvDD-treated tumors than vvDD-IL-23-treated tumors at day 9 after treatment (Figure 4B). This suggests a sustainable GzmB expression induced by IL-23. The expression pattern of immune checkpoints CTLA-4, PD-1 and PD-L1 were increased at day 5 after both viral treatments, but remained higher after vvDD-IL-23 treatment at day 9, similar to the patterns of perforin and IL-2 (Figure 4C). The pro-tumor factors CCL22 and IDO1 expression did not significantly change at either day 5 or day 9 after either viral treatments compared with PBS treatment (Figure S3), while the pro-tumor factor COX2 was significantly increased at day 5, but not at day 9 after vvDD-IL-23 treatment (Figure S3), which might contribute to the decrease of both G-MDSC and M-MDSC in tumor CD45^+^CD11b^+^ cells at day 9 (Figure 4D) since the COX2-PGE2 (prostaglandin E2) feedback plays an important role in the induction and persistence of MDSCs 41.

In summary, both viral infection and IL-23 delivery elevated IL-23R and IL-10 expression, which might prolong the viral persistence of vvDD-IL-23 in the TME and, in turn, lead to more IL-23 expression. This accumulated IL-23 might further modulate the TME to be more conducive to antitumor immunity via elevated Th1 chemokines and antitumor factors, leading to an improved antitumor effect.

### IL-23 expressing oncolytic virus elicits antitumor effects in late-stage tumor models

**Figure 5:**
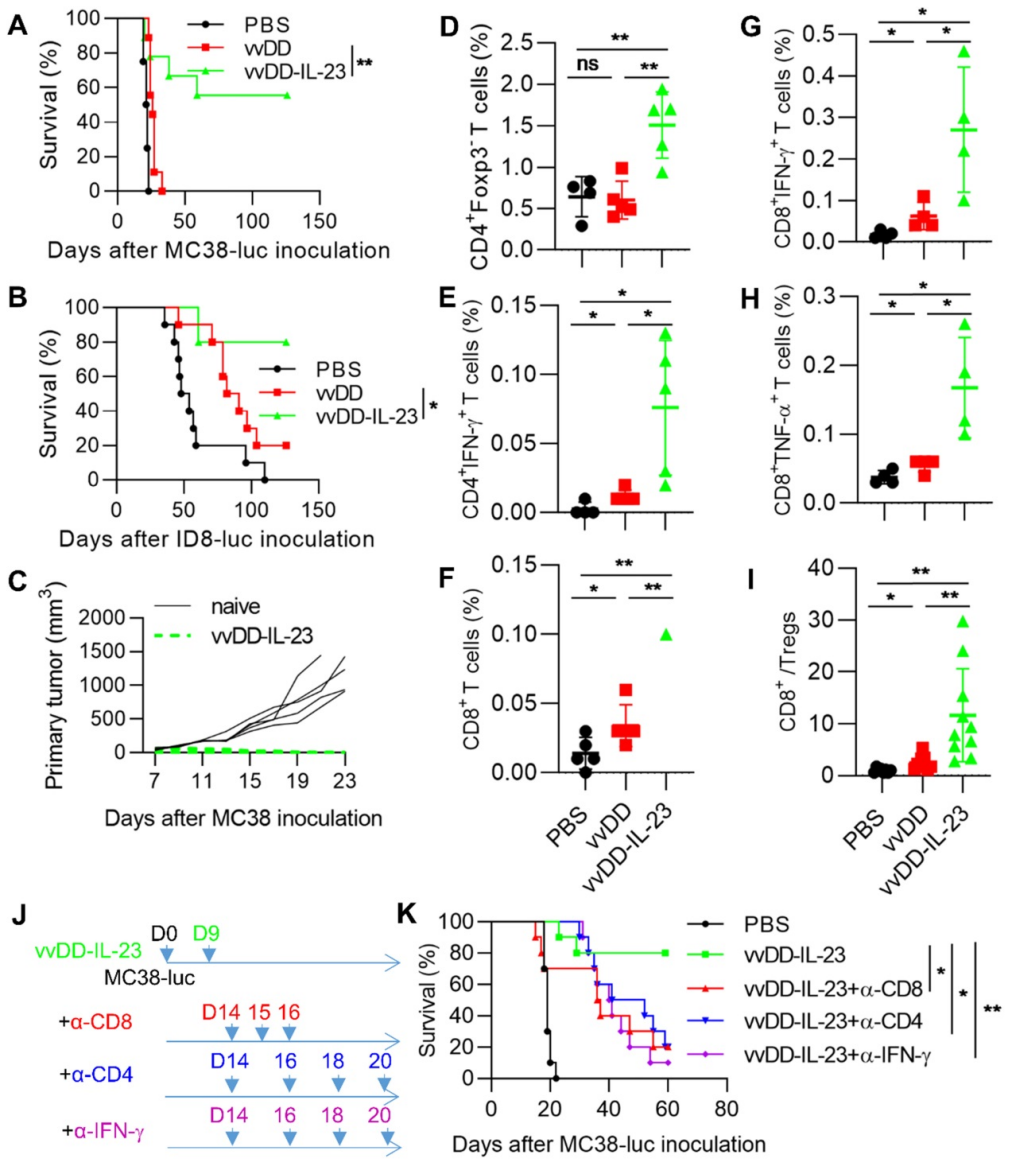
** vvDD-IL-23 treatment elicits potent therapeutic effects in late-stage tumor models.** B6 mice were i.p. inoculated with 5×10^5^ MC38-luc or 3.5×10^6^ ID8-luc cells and treated with PBS, vvDD, or vvDD-IL-23 at 2×10^8^ PFU/mouse nine days after tumor inoculation, and the survival curves are shown in (**A**) and (**B**), respectively. (**C**) Naïve B6 mice or MC38-luc-tumor-bearing B6 mice treated with vvDD-IL-23, which had survived for more than 120 days, were s.c. challenged with 1×10^6^ MC38 cells. The tumor growth curve is shown. B6 mice were i.p. inoculated with 5×10^5^ MC38-luc cells and treated with PBS, vvDD, or vvDD-IL-23 at 2×10^8^ PFU/mouse nine days after tumor inoculation. Tumor-bearing mice were sacrificed five days after treatment and primary tumors were collected and analyzed using flow cytometry to determine CD4^+^Foxp3^-^ (**D**), CD4^+^IFN-γ^+^ (**E**), CD8^+^ (**F**), CD8^+^IFN-γ^+^ (**G**), CD8^+^TNF-α^+^(**H**) T cells in tumors and CD8^+^/Treg ratio (**I**). In a separate experiment, B6 mice were i.p. inoculated with 5×10^5^ MC38-luc cells and treated with vvDD-IL-23 nine days after tumor inoculation. α-CD8 Ab (250 µg per injection), α-CD4 Ab (150 µg per injection), or α-IFN-γ Ab (200 µg per injection) were i.p. injected into mice to deplete CD8^+^ T cells, CD4^+^ T cells, or neutralize circulating IFN-γ, respectively (**J**), and a log-rank (Mantel-Cox) test was used to compare survival rates (**K**). *: *P*<0.05; **: *P*<0.01. ns: not significant.

To explore whether vvDD-IL-23 could induce an antitumor effect in late-stage tumor models, B6 mice were i.p. injected with MC38-luc or ID8-luc and these tumor-bearing mice were treated 9 days after tumor cell inoculation. The mice receiving vvDD-IL-23 treatment survived significantly longer than other treatments in both tumor models and cure rates were 55.6% (five out of nine MC-38-luc-bearing mice were cured) and 80% (eight out of ten ID-8-luc-bearing mice were cured), respectively (Figure 5A-B). The MC38-luc-bearing mice which were cured by vvDD-IL-23 treatment were s.c. challenged with a high dose of MC38 (1×10^6^ per mouse). All these mice rejected the challenge (Figure 5C), indicating an established systemic antitumor immunity in these mice. We also measured functional T cells in the TME. CD4^+^Foxp3^-^, CD4^+^IFN-γ^+^, CD8^+^, CD8^+^IFN-γ^+^ and CD8^+^TNF-α^+^ T cells in tumors were significantly elevated after vvDD-IL-23 treatment compared with other treatments (Figure 5D-H). Though vvDD-IL-23 treatment did not change CD4^+^Foxp3^+^ T cells (Tregs, Regulatory T cells) (Figure S4), the ratio of CD8^+^/Treg was significantly increased (Figure 5I), suggesting a transformation of “cold” tumors into “hot” tumors. We further depleted IFN-γ, CD4^+^ T cells and CD8^+^ T cells by antibodies after vvDD-IL-23 treatment (Figure 5J) and found that the antitumor effect elicited by vvDD-IL-23 treatment was IFN-γ- CD8^+^ T cell-, and CD4^+^ T cell-dependent (Figure 5K). Collectively, these data demonstrate that vvDD-IL-23 treatment elicited potent systemic antitumor effects in late-stage tumor models and the antitumor effect is IFN-γ- CD8^+^ T cell- and CD4^+^ T cell-dependent.

### IL-23A expressing oncolytic virus cannot elicit antitumor effects

**Figure 6:**
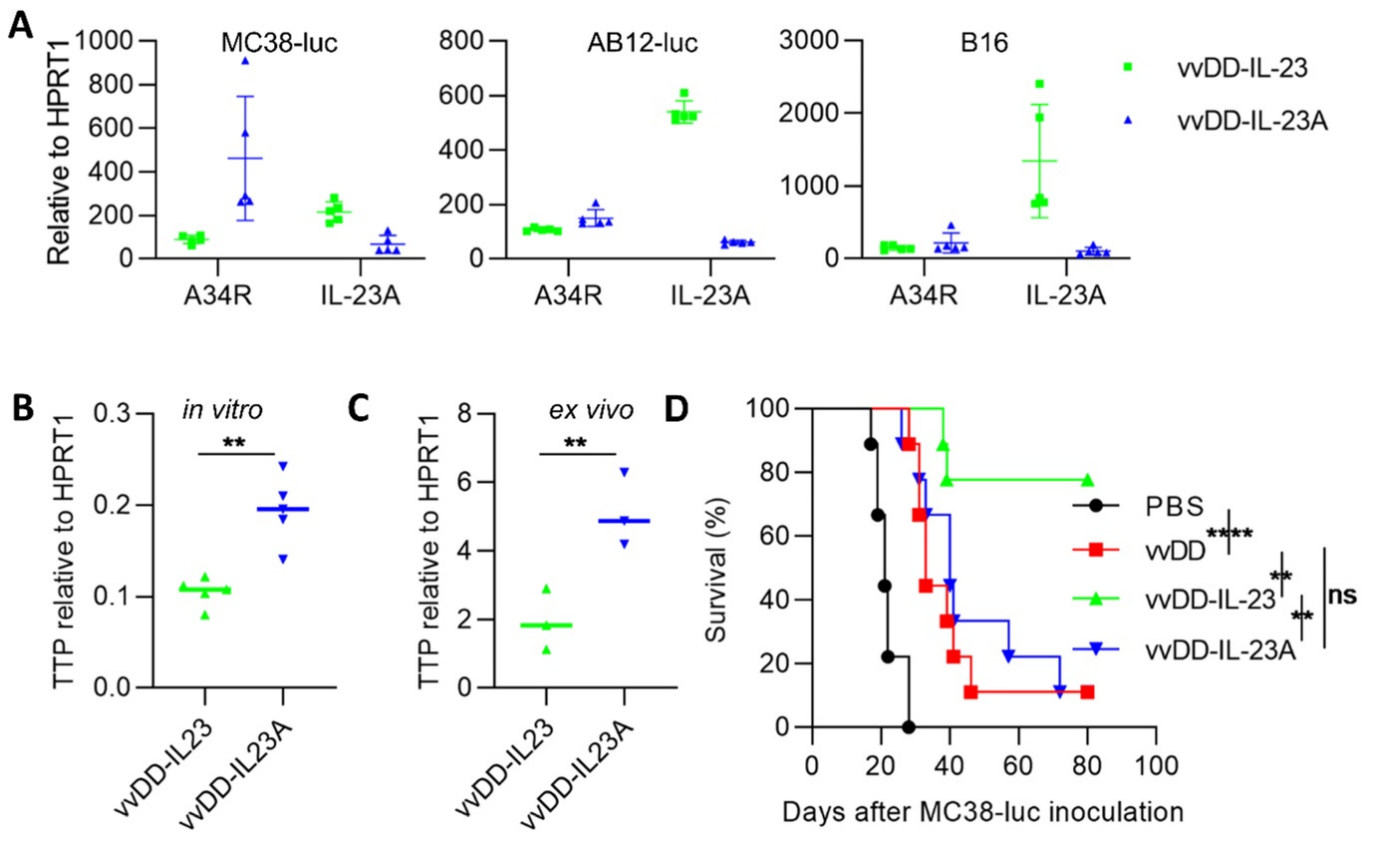
** vvDD-IL-23A treatment cannot modulate therapeutic effects *in vivo*.** (**A**) MC38-luc (3×10^5^ cells), AB12-luc (3×10^5^ cells) or B16 (2×10^5^ cells) tumor cells were infected with vvDD-IL-23 or vvDD-IL-23A at an MOI of 1. The cell pellets were harvested to measure A34R or IL-23 expression at 24 h using RT-qPCR. (**B**) MC38-luc (3×10^5^ cells) tumor cells were infected with vvDD-IL-23 or vvDD-IL-23A at an MOI of 1. The cell pellets were harvested to determine TTP expression at 24 h using RT-qPCR. (**C**) B6 mice were i.p. inoculated with 5×10^5^ MC38-luc cells and treated with vvDD-IL-23 or vvDD-IL-23A at 2×10^8^ PFU/mouse five days after tumor inoculation. Tumor-bearing mice were sacrificed nine days after treatment and primary tumors were collected and analyzed using RT-qPCR to determine TTP expression using RT-qPCR. (**D**) B6 mice were i.p. inoculated with 5×10^5^ MC38-luc cells and treated with PBS, vvDD, vvDD-IL-23 or vvDD-IL-23A at 2×10^8^ PFU/mouse five days after tumor inoculation, and a log-rank (Mantel-Cox) test was used to compare survival rates. *: *P*<0.05; **: *P*<0.01; and ****: *P*<0.0001. ns: not significant.

Other investigators demonstrated that a subunit of the IL-23 heterodimeric cytokine, encoded by the *il-23a* gene, significantly promoted tumor growth and shortened survival when integrated into 4T1 cells 37. We asked whether viral delivered IL-23A could promote or suppress tumor growth. We generated IL-23A-expressing vvDD-IL-23A (identified by PCR amplification of *il-23a* cDNA from viral DNA; data not shown) and measured its expression in tumor cells *in vitro*. We were surprised to find that IL-23A mRNA from vvDD-IL-23A-infected MC38-luc was significantly lower than that from vvDD-IL-23-infected MC38-luc, though viral replication (A34R expression) was comparable. And this was further repeated in AB12-luc and B16 cells (Figure 6A). Tristetraprolin (TTP), also known as zinc finger protein 36 homolog, was suggested to strongly decrease IL-23 production and IL-23A mRNA stability 42. We tested TTP expression using MC38 tumor model. We found that the mRNA of TTP was significantly higher in vvDD-IL-23A-treated tumor cells (Figure 6B) or tumor tissue (Figure 6C) compared with vvDD-IL-23-treated tumor cells (Figure 6B) or tumor tissue (Figure 6C), indicating that TTP may negatively impact the IL-23A mRNA stability after vvDD-IL-23A infection, preventing its pro-tumor or antitumor activity (Figure 6D).

### Membrane-bound IL-23 expressing oncolytic virus elicits antitumor effects

**Figure 7:**
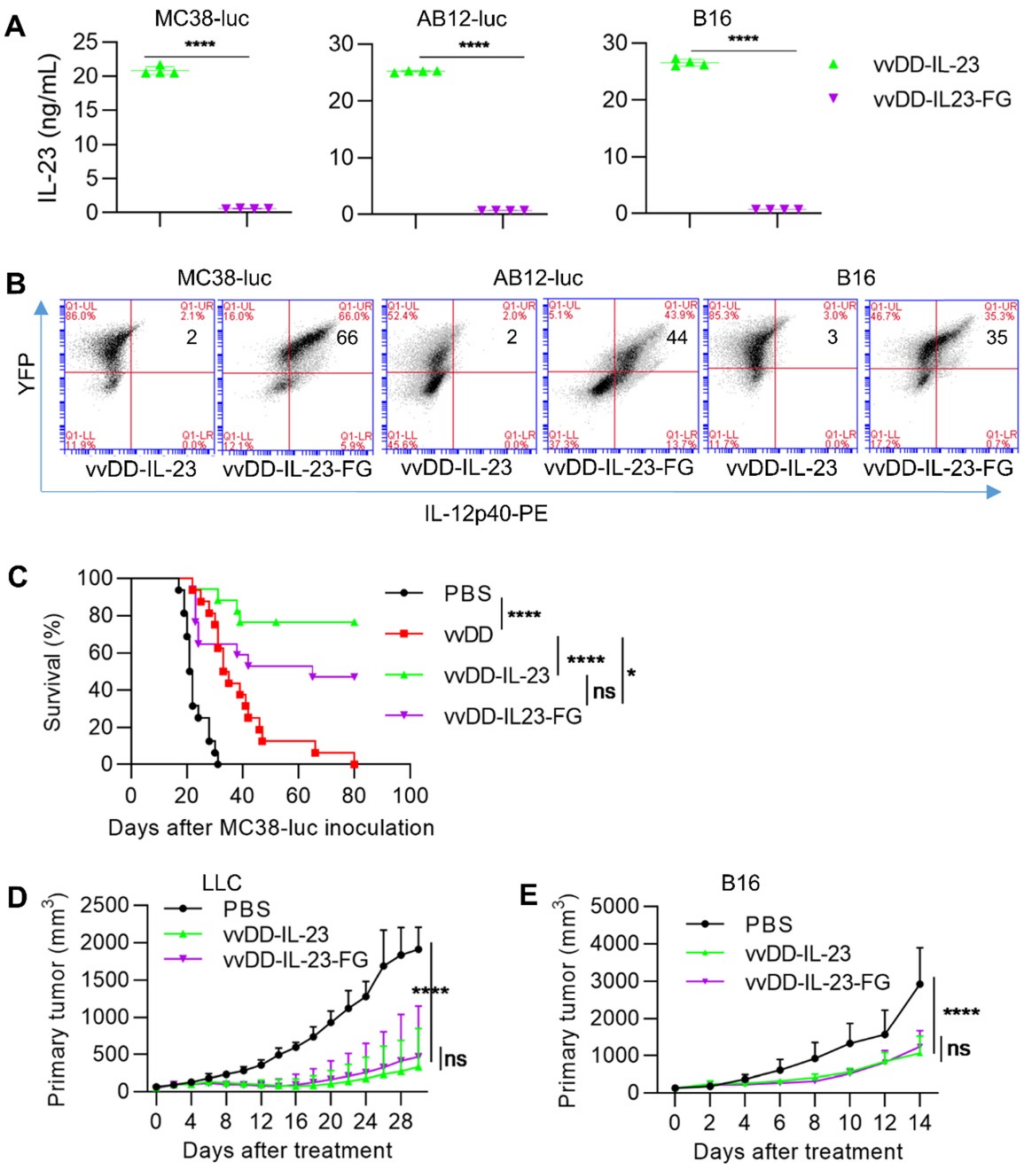
** vvDD-IL-23-FG treatment elicits therapeutic effects *in vivo*.** MC38-luc (3×10^5^ cells), AB12-luc (3×10^5^ cells) or B16 (2×10^5^ cells) tumor cells were infected with vvDD-IL-23 or vvDD-IL-23A at an MOI of 1. Twenty-four h after infection, the supernatants were harvested to measure IL-23 using ELISA (**A**) and the cell pellets were harvested to measure membrane-bound IL-23 using flow cytometry (**B**). (**C**) B6 mice were i.p. inoculated with 5×10^5^ MC38-luc cells and treated with PBS, vvDD, vvDD-IL-23 or vvDD-IL-23-FG at 2×10^8^ PFU/mouse five days after tumor inoculation, and a log-rank (Mantel-Cox) test was used to compare survival rates. B6 mice were s.c. inoculated with 1×10^6^ LLC or 2×10^5^ B16 in the right flanks, and were i.t. treated with 60 µL PBS or 5×10^7^ PFU/60 µL virus per mouse when tumor volume reached 50 mm^3^ or 140 mm^3^, and tumor growth curves are shown in (**D**) and (**E**), respectively. The endpoints were determined by natural death or tumor size over 2 cm. *: *P*<0.05; ****: *P*<0.0001. ns: not significant.

Systemic IL-23 application did not induce intense toxicity as has been seen with IL-12 43; however, oncolytic vaccinia virus can replicate robustly in the TME and accumulate transgene products in a short time, especially in the late-stage tumors with high tumor volume, and thus, the possible toxicity might be considered. We constructed a membrane-bound IL-23 expressing oncolytic vaccinia virus vvDD-IL-23-FG using a glycosylphosphatidylinositol (GPI) anchor form of human CD16b to fuse to IL-12p40 via a flexible linker (G_4_S)_3_ so as to tether IL-23 on the cell membrane (Figure S1). When MC38-luc, AB12-luc, and B16 cells were infected with these two IL-23-armed viruses at an MOI of 1, mRNA levels of viral housekeeping gene (A34R) and IL-12p40 were similar (data not shown). We also measured IL-23 expression at the protein level using ELISA and flow cytometry. The amount of IL-23 in the supernatant from vvDD-IL-23-infected tumor cells was significantly higher than vvDD-IL-23-FG (Figure 7A), while IL-23^+^ cells were significantly more prevalent in vvDD-IL-23-FG-infected cells (Figure 7B), showing the successful realization of membrane association by GPI anchored to one subunit of IL-23. Notably, both viruses have YFP as a marker and their infections were similar, evidencing by the similar YFP^+^ cells after infection (Figure 7B). We then evaluated the antitumor efficacy of these two IL-23-expressing viruses. B6 mice bearing five-day old peritoneal murine colon cancer MC38-luc were i.p. injected with PBS, vvDD, vvDD-IL-23, or vvDD-IL-23-FG at the dose of 2×10^8^ PFU per mouse. The survival results demonstrated that both vvDD-IL-23 and vvDD-IL-23-FG elicited significantly potent antitumor effects compared with PBS or vvDD treatment, and the antitumor effect induced by both IL-23-expressing viruses was not significantly different (Figure 7C). We further evaluated their antitumor effects on the LLC subcutaneous model and a late-stage B16 subcutaneous model (average tumor volume equals 140 mm^3^ when treatments started). The tumor growth curves were comparable after vvDD-IL-23 or vvDD-IL-23-FG treatment (Figure 7D-E). While no early deaths were observed in any of these models after vvDD-IL-23 treatment, potential long-term toxicities associated with the IL-23 virus have not been assessed in these models, and a toxicology study would need to be performed prior to clinical trials.

## Discussion

Cancer immunotherapy has joined with conventional cancer therapies as a successful modality to treat cancer. “Hot” or “inflamed” tumors are more responsive to immunotherapy approaches. However, the majority of solid tumors are defined as “cold” or “immune-desert” tumors 2-4. Therefore, new approaches that can improve T cell infiltration and tip the cancer-immune set point in the TME 2, resulting in “hot” or T cell-inflamed tumors, are urgently needed to improve the therapeutic efficacy and application of cancer immunotherapy. Infectious disease vaccines were repurposed for cancer immunotherapy owing to their abilities to induce potent antitumor immune responses 44. Replication-competent oncolytic viruses are among these vaccines and can exert antitumor responses beyond their oncolytic nature, especially when these viruses were armed with genes such as cytokines, chemokines, and costimulatory molecules in order to augment antitumor immunity 5, 7, 9-11. Thus, oncolytic virus can be applied as a cancer immunotherapy approach either alone or in combination with other cancer immunotherapies 13, 14.

The IL-12 cytokine family includes four heterodimeric members: IL-12, IL-23, IL-27 and IL-35. While IL-12 functions undoubtedly to enhance antitumor effects 32, IL-23 is known to have conflicting roles in cancer development and treatment. Both IL-23A and IL-23R deficient mice were found to be resistant to chemical-induced skin papillomas or fibrosarcomas. Anti-IL-23A monoclonal antibody treatment synergistically suppressed tumor growth and metastases in combination with either targeted therapies or IL-2. However, various IL-23-overexpressing murine cancer cells, such as colon cancer, melanoma, fibrosarcoma, mammary carcinoma, glioma and hepatocellular carcinoma, impaired tumor growth *in vivo* compared with wild type tumor cells 33. IL-23-expressing adenovirus or vesicular stomatitis virus also induced antitumor effects compared with wild type viruses 34-36. Here, we used oncolytic vaccinia virus as a platform to deliver IL-23 into the tumor bed and investigated the TME. In this MC38 model, IL-23 expression prolonged the viral persistence, possibly mediated by up-regulated IL-10 in the TME, as IL-10 was previously applied to arm vaccinia virus to achieve an extended viral persistence 38 and in this previous study, IL-10 decreased macrophage infiltration and downregulated MHCII expression, leading to a delay in immune clearance, while not directly affecting the replication or spread of the virus. The extended persistence of vvDD-IL-23 resulted in sustained IL-23 expression and IL-23 accumulation. The IL-23 plus viral oncolysis further elevated Th1 chemokines and antitumor factors such as IFN-γ, TNF-α, IL-2, Perforin and GzmB, increased infiltrating activated T cells and the CD8^+^/Treg ratio, and finally transformed the immune-suppressive TME and exerted a potent antitumor effect. It is worth mentioning that the virus in the tumor treated with vvDD-IL-23 was mostly cleared by D11 after treatment, suggesting the long-term safety of vvDD-IL-23 application. Notably, investigators have previously demonstrated that low concentrations of IL-23 bind to IL-23R and promote lung cancer cell growth, whereas high concentrations of IL-23 binds to both IL-23R and IL-12β1 to inhibit lung cancer growth 45. Thus, accumulated IL-23 in our model might also directly inhibit tumor growth. The viral treatment induced increased IL-23R expression both *in vitro* and *in vivo*, suggesting the rational combination of oncolytic vaccinia virus and IL-23 expression. The vvDD-IL-23 treatment also elevated COX-2 and immune checkpoint expression in the TME, indicating a rational combination with COX-2 inhibitors and immune checkpoint blockade in the future. Anti-CTLA-4 and anti-PD-1/PD-L1 antibodies can induce immune-related adverse events (irAEs). The combination of vvDD-IL-23 and immune checkpoint blockade might induce autoimmune diseases since IL-23 promotes the development of an IL-17-producing CD4^+^ helper T cell subset. However, we did not detect significant IL-17 expression in virus-treated tumors (data not shown), which might be attributed to the suppression by elevated IL-10 expression. IL-10 was previously reported to suppress IL-17 expression 46, 47. Also of note, IL-17 might function little in vvDD-IL-23 mediated antitumor effects since it was reported that IL-23 delivered by adenovirus can elicit antitumor effects in IL-17R knockout mice 36. Neutralizing antibodies play an important role in the activity of oncolytic viruses, especially limiting repeat injections 48, 49. While beyond the scope of the current study, the effect of IL-23 expression on antibody formation should be examined in the future.

Recently, murine breast cancer cells (4T1) were used to generate a stable cell line expressing IL-23A. This IL-23A overexpression promoted tumor growth by increasing the infiltration of M2 macrophage and neutrophils and the secretion of immunosuppressive cytokines in the TME 37. To the best of our knowledge, this is the first report showing that the overexpressing IL-23A could promote tumor growth. We asked whether IL-23A-expressing vaccinia virus vvDD-IL-23A could promote or inhibit tumor growth. Our results showed that vvDD-IL-23A did not promote or inhibit tumor growth compared with its parental virus vvDD. This phenomenon might be attributed to the instability of IL-23A mRNA due to high TTP which can destabilize IL-23A mRNA 42 after vvDD-IL-23A treatment, compared with vvDD-IL-23 treatment. We postulate that over-accumulated IL-23A might induce TTP synthesis so as to decrease the accumulation of IL-23A. However, when IL-23A engages with IL-12p40 to form a biologically functional IL-23, it loses the trigger to up-regulate TTP synthesis and is protected from degradation. The exact mechanism needs further investigation. It is worth mentioning that IL-23A may have pro-tumor activity that is not appreciated in the context of a replicating virus delivery.

Toxicity related to cytokine release syndrome should be tightly controlled in any cytokine- or cell-based immunotherapy. We have successfully managed the severe life-threatening side effects associated with systemic IL-2 or IL-12 application while maintaining their therapeutic functions in the TME using viral delivered membrane-bound forms 27, 31. Though the side effects associated with systemic IL-23 application is not as severe as IL-12 43, in our models, the rapid replication of oncolytic vaccinia virus may lead to accumulation of a large amount of IL-23 which might surpass the previously tested dose in a short time, leading to severe toxicity. Thus, we constructed and tested a membrane-bound IL-23-expressing virus, vvDD-IL-23-FG. The two kinds of IL-23-expressing viruses elicited similar therapeutic effects on two subcutaneous tumor models, but vvDD-IL-23-FG elicited weaker antitumor effects on the intraperitoneal MC38 tumor model, though this difference is not statistically significant. The reason for this differential effect needs further investigation. While we have not investigated specifically IL-23R binding to tethered IL-23, the positive antitumor, immunologic effect suggests that it is active. We have demonstrated activity of tethered IL-2 and IL-12 in previous studies *in vitro* using T-cell proliferation assays 27, 31.

## Conclusion

Our data demonstrate that vvDD-IL-23 treatment can deliver IL-23, not IL-23A to the tumor bed and elevate IL-10 expression and, in turn, prolong viral persistence and IL-23 accumulation. The viral oncolysis and IL-23 expression transform the immunosuppressive TME and exert potent antitumor effects. Membrane-bound IL-23 can function with viral delivery, suggesting a feasible approach to manage possible side effects associated with IL-23 application. Our data suggest that IL-23 might be worth revisiting as a candidate for oncolytic virus based cancer immunotherapy.

## Supplementary Material

Supplementary figures.

## References

[1] Groeneveldt C, van Hall T, van der Burg SH, Ten Dijke P, van Montfoort N (2020). Immunotherapeutic Potential of TGF-beta Inhibition and Oncolytic Viruses. Trends Immunol.

[2] Chen DS, Mellman I (2017). Elements of cancer immunity and the cancer-immune set point. Nature.

[3] Gajewski TF (2015). The Next Hurdle in Cancer Immunotherapy: Overcoming the Non-T-Cell-Inflamed Tumor Microenvironment. Semin Oncol.

[4] van der Woude LL, Gorris MAJ, Halilovic A, Figdor CG, de Vries IJM (2017). Migrating into the Tumor: a Roadmap for T Cells. Trends Cancer.

[5] Bartlett DL, Liu Z, Sathaiah M, Ravindranathan R, Guo Z, He Y (2013). Oncolytic viruses as therapeutic cancer vaccines. Mol Cancer.

[6] Guo ZS, Lu B, Guo Z, Giehl E, Feist M, Dai E (2019). Vaccinia virus-mediated cancer immunotherapy: cancer vaccines and oncolytics. J Immunother Cancer.

[7] Bommareddy PK, Shettigar M, Kaufman HL (2018). Integrating oncolytic viruses in combination cancer immunotherapy. Nat Rev Immunol.

[8] Twumasi-Boateng K, Pettigrew JL, Kwok YYE, Bell JC, Nelson BH (2018). Oncolytic viruses as engineering platforms for combination immunotherapy. Nat Rev Cancer.

[9] Guo ZS, Liu Z, Kowalsky S, Feist M, Kalinski P, Lu B (2017). Oncolytic Immunotherapy: Conceptual Evolution, Current Strategies, and Future Perspectives. Front Immunol.

[10] Harrington K, Freeman DJ, Kelly B, Harper J, Soria JC (2019). Optimizing oncolytic virotherapy in cancer treatment. Nat Rev Drug Discov.

[11] Pelin A, Boulton S, Tamming LA, Bell JC, Singaravelu R (2020). Engineering vaccinia virus as an immunotherapeutic battleship to overcome tumor heterogeneity. Expert Opin Biol Ther.

[12] Murciano-Goroff YR, Warner AB, Wolchok JD (2020). The future of cancer immunotherapy: microenvironment-targeting combinations. Cell Res.

[13] Kaufman HL, Kohlhapp FJ, Zloza A (2015). Oncolytic viruses: a new class of immunotherapy drugs. Nat Rev Drug Discov.

[14] Hemminki O, Dos Santos JM, Hemminki A (2020). Oncolytic viruses for cancer immunotherapy. J Hematol Oncol.

[15] Samson A, Scott KJ, Taggart D, West EJ, Wilson E, Nuovo GJ (2018). Intravenous delivery of oncolytic reovirus to brain tumor patients immunologically primes for subsequent checkpoint blockade. Sci Transl Med.

[16] Bourgeois-Daigneault MC, Roy DG, Aitken AS, El Sayes N, Martin NT, Varette O (2018). Neoadjuvant oncolytic virotherapy before surgery sensitizes triple-negative breast cancer to immune checkpoint therapy. Sci Transl Med.

[17] Zamarin D, Holmgaard RB, Subudhi SK, Park JS, Mansour M, Palese P (2014). Localized oncolytic virotherapy overcomes systemic tumor resistance to immune checkpoint blockade immunotherapy. Sci Transl Med.

[18] Smith HG, Mansfield D, Roulstone V, Kyula-Currie JN, McLaughlin M, Patel RR (2019). PD-1 Blockade Following Isolated Limb Perfusion with Vaccinia Virus Prevents Local and Distant Relapse of Soft-tissue Sarcoma. Clin Cancer Res.

[19] Chon HJ, Lee WS, Yang H, Kong SJ, Lee NK, Moon ES (2019). Tumor Microenvironment Remodeling by Intratumoral Oncolytic Vaccinia Virus Enhances the Efficacy of Immune-Checkpoint Blockade. Clin Cancer Res.

[20] Liu Z, Ravindranathan R, Li J, Kalinski P, Guo ZS, Bartlett DL (2016). CXCL11-Armed oncolytic poxvirus elicits potent antitumor immunity and shows enhanced therapeutic efficacy. Oncoimmunology.

[21] Kowalsky SJ, Liu Z, Feist M, Berkey SE, Ma C, Ravindranathan R (2018). Superagonist IL-15-Armed Oncolytic Virus Elicits Potent Antitumor Immunity and Therapy That Are Enhanced with PD-1 Blockade. Mol Ther.

[22] Cervera-Carrascon V, Siurala M, Santos JM, Havunen R, Tahtinen S, Karell P (2018). TNFa and IL-2 armed adenoviruses enable complete responses by anti-PD-1 checkpoint blockade. Oncoimmunology.

[23] Liu Z, Ravindranathan R, Kalinski P, Guo ZS, Bartlett DL (2017). Rational combination of oncolytic vaccinia virus and PD-L1 blockade works synergistically to enhance therapeutic efficacy. Nat Commun.

[24] Ribas A, Dummer R, Puzanov I, VanderWalde A, Andtbacka RHI, Michielin O (2017). Oncolytic Virotherapy Promotes Intratumoral T Cell Infiltration and Improves Anti-PD-1 Immunotherapy. Cell.

[25] Saha D, Martuza RL, Rabkin SD (2017). Macrophage Polarization Contributes to Glioblastoma Eradication by Combination Immunovirotherapy and Immune Checkpoint Blockade. Cancer Cell.

[26] Zamarin D, Holmgaard RB, Ricca J, Plitt T, Palese P, Sharma P (2017). Intratumoral modulation of the inducible co-stimulator ICOS by recombinant oncolytic virus promotes systemic anti-tumour immunity. Nat Commun.

[27] Liu Z, Ge Y, Wang H, Ma C, Feist M, Ju S (2018). Modifying the cancer-immune set point using vaccinia virus expressing re-designed interleukin-2. Nat Commun.

[28] McCart JA, Ward JM, Lee J, Hu Y, Alexander HR, Libutti SK (2001). Systemic cancer therapy with a tumor-selective vaccinia virus mutant lacking thymidine kinase and vaccinia growth factor genes. Cancer Res.

[29] Zeh HJ, Downs-Canner S, McCart JA, Guo ZS, Rao UN, Ramalingam L (2015). First-in-man study of western reserve strain oncolytic vaccinia virus: safety, systemic spread, and antitumor activity. Mol Ther.

[30] Downs-Canner S, Guo ZS, Ravindranathan R, Breitbach CJ, O'Malley ME, Jones HL (2016). Phase 1 Study of Intravenous Oncolytic Poxvirus (vvDD) in Patients With Advanced Solid Cancers. Mol Ther.

[31] Ge Y, Wang H, Ren J, Liu W, Chen L, Chen H (2020). Oncolytic vaccinia virus delivering tethered IL-12 enhances antitumor effects with improved safety. J Immunother Cancer.

[32] Tugues S, Burkhard SH, Ohs I, Vrohlings M, Nussbaum K, Vom Berg J (2015). New insights into IL-12-mediated tumor suppression. Cell Death Differ.

[33] Ngiow SF, Teng MW, Smyth MJ (2013). A balance of interleukin-12 and -23 in cancer. Trends Immunol.

[34] Miller JM, Bidula SM, Jensen TM, Reiss CS (2010). Vesicular stomatitis virus modified with single chain IL-23 exhibits oncolytic activity against tumor cells *in vitro* and *in vivo*. Int J Interferon Cytokine Mediat Res.

[35] Choi IK, Li Y, Oh E, Kim J, Yun CO (2013). Oncolytic adenovirus expressing IL-23 and p35 elicits IFN-gamma- and TNF-alpha-co-producing T cell-mediated antitumor immunity. PLoS One.

[36] Reay J, Kim SH, Lockhart E, Kolls J, Robbins PD (2009). Adenoviral-mediated, intratumor gene transfer of interleukin 23 induces a therapeutic antitumor response. Cancer Gene Ther.

[37] Nie W, Yu T, Sang Y, Gao X (2017). Tumor-promoting effect of IL-23 in mammary cancer mediated by infiltration of M2 macrophages and neutrophils in tumor microenvironment. Biochem Biophys Res Commun.

[38] Chard LS, Maniati E, Wang P, Zhang Z, Gao D, Wang J (2015). A vaccinia virus armed with interleukin-10 is a promising therapeutic agent for treatment of murine pancreatic cancer. Clin Cancer Res.

[39] Vanden Eijnden S, Goriely S, De Wit D, Willems F, Goldman M (2005). IL-23 up-regulates IL-10 and induces IL-17 synthesis by polyclonally activated naive T cells in human. Eur J Immunol.

[40] Sun R, Hedl M, Abraham C (2020). IL23 induces IL23R recycling and amplifies innate receptor-induced signalling and cytokines in human macrophages, and the IBD-protective IL23R R381Q variant modulates these outcomes. Gut.

[41] Obermajer N, Muthuswamy R, Lesnock J, Edwards RP, Kalinski P (2011). Positive feedback between PGE2 and COX2 redirects the differentiation of human dendritic cells toward stable myeloid-derived suppressor cells. Blood.

[42] Molle C, Zhang T, Ysebrant de Lendonck L, Gueydan C, Andrianne M, Sherer F (2013). Tristetraprolin regulation of interleukin 23 mRNA stability prevents a spontaneous inflammatory disease. J Exp Med.

[43] Oniki S, Nagai H, Horikawa T, Furukawa J, Belladonna ML, Yoshimoto T (2006). Interleukin-23 and interleukin-27 exert quite different antitumor and vaccine effects on poorly immunogenic melanoma. Cancer Res.

[44] Melero I, Gato M, Shekarian T, Aznar A, Valsesia-Wittmann S, Caux C (2020). Repurposing infectious disease vaccines for intratumoral immunotherapy. J Immunother Cancer.

[45] Li J, Zhang L, Zhang J, Wei Y, Li K, Huang L (2013). Interleukin 23 regulates proliferation of lung cancer cells in a concentration-dependent way in association with the interleukin-23 receptor. Carcinogenesis.

[46] Gu Y, Yang J, Ouyang X, Liu W, Li H, Yang J (2008). Interleukin 10 suppresses Th17 cytokines secreted by macrophages and T cells. Eur J Immunol.

[47] Vultaggio A, Nencini F, Pratesi S, Maggi L, Guarna A, Annunziato F (2011). The TLR7 ligand 9-benzyl-2-butoxy-8-hydroxy adenine inhibits IL-17 response by eliciting IL-10 and IL-10-inducing cytokines. J Immunol.

[48] Magge D, Guo ZS, O'Malley ME, Francis L, Ravindranathan R, Bartlett DL (2013). Inhibitors of C5 complement enhance vaccinia virus oncolysis. Cancer Gene Ther.

[49] Guo ZS, Thorne SH, Bartlett DL (2008). Oncolytic virotherapy: molecular targets in tumor-selective replication and carrier cell-mediated delivery of oncolytic viruses. Biochim Biophys Acta.

