# Supplementary material for: Intratumoral expression of interleukin 23 variants using oncolytic vaccinia virus elicit potent antitumor effects on multiple tumor models via tumor microenvironment modulation
Source: Theranostics. 2021 May 3;11(14):6668–81. doi: 10.7150/thno.56494 (PMC8171085; doi:10.7150/thno.56494)
Supplement: Supplementary file 1 — Supplementary figures. [file thnov11p6668s1.pdf]

**Intratumoral expression of interleukin 23 variants using oncolytic vaccinia virus elicit potent antitumor effects on multiple tumor models via tumor microenvironment modulation**

**Lingjuan Chen[1-4,#], Hongqi Chen[3,5#], Junjie Ye[1-3,6#], Yan Ge[3,7], Haiyan Wang[3,7], Enyong Dai[3,8], Jinghua Ren[3,4], Weilin Liu[3,9], Congrong Ma[3], Songguang Ju[7], Z. Sheng Guo[3], Zuqiang Liu[1-3]*, and David L. Bartlett[1-3]***

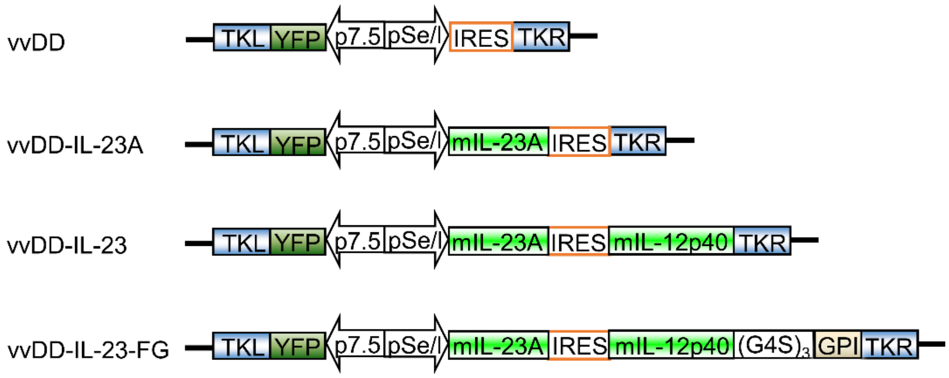


**Figure S1. Schematic diagram of viral IL-23 variants.** vvDD-IL-23A, vvDD-IL-23, and vvDD-IL-23-FG were generated by homologous recombination of murine IL-23 variants into the tk locus of vaccinia viral genome of VSC20, carrying IL-23A, IL-23, and IL-23-flexible linker (G4S)3-GPI anchor sequence amplified from human CD16b, respectively.

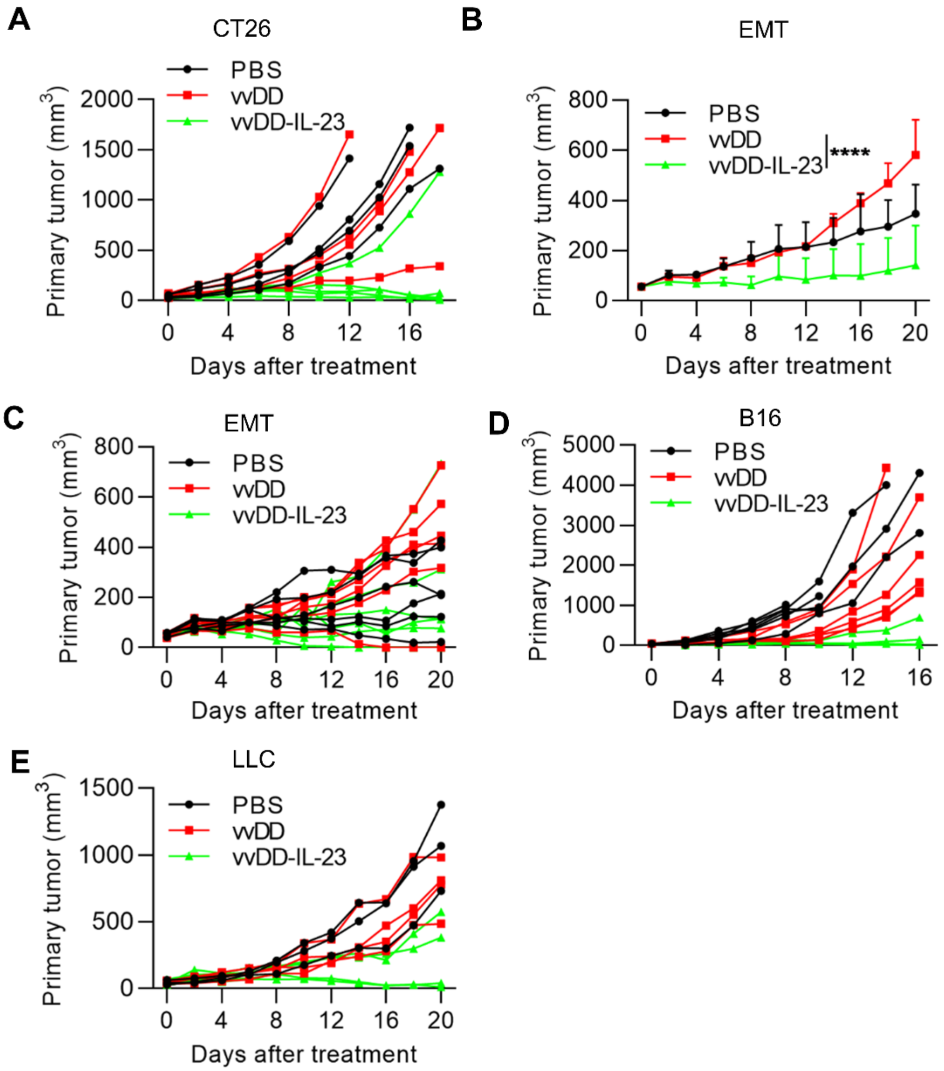


**Figure S2. vvDD-IL-23 treatment elicits potent therapeutic effects in subcutaneous tumor models.** BalB/c mice were s.c. inoculated with $1\times10^6$ CT26 (**A**) in the right flank or $1\times10^6$ EMT6 (**B-C**) in the mammary fat pad or B6 mice were s.c. inoculated with $2\times10^5$ B16 (**D**) or $5\times10^5$ LLC (**E**) in the right flank. The resulting tumor-bearing mice were i.t. treated with 60 µL PBS or $5\times10^7$ PFU/60 µL virus per mouse at day 6 (CT26 and EMT6), 10 (B16) or 7 (LLC) after tumor cell inoculation, respectively. Tumor growth curves are shown, respectively. A two-way ANOVA test was used to compare tumor growth cures. ****: $P<0.0001$.

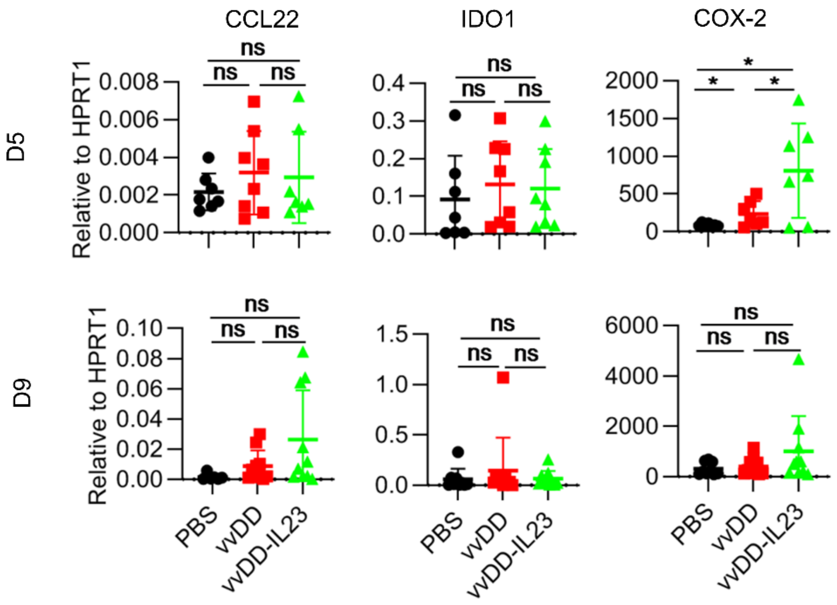


**Figure S3. vvDD-IL-23 treatment transforms TME.** B6 mice were i.p. inoculated with $5\times10^5$ MC38-luc cells and treated with PBS, vvDD, or vvDD-IL-23 at $2\times10^8$ PFU/mouse five days after tumor inoculation. Tumor-bearing mice were sacrificed five or nine days after treatment and primary tumors were collected and analyzed using RT-qPCR to determine the expression of CCL22, IDO1 and COX-2 in the TME. *: $P<0.05$. ns: not significant.

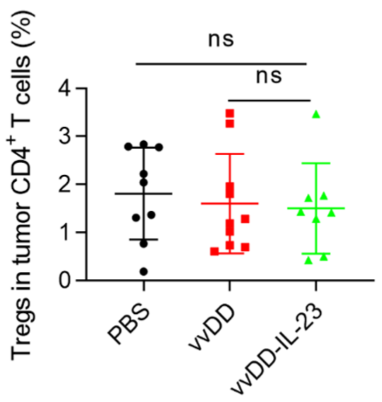


**Figure S4. vvDD-IL-23 treatment does not increase Treg accumulation in TME.** B6 mice were i.p. inoculated with $5\times10^5$ MC38-luc cells and treated with PBS, vvDD, or vvDD-IL-23 at $2\times10^8$ PFU/mouse nine days after tumor inoculation. Tumor-bearing mice were sacrificed five days after treatment and primary tumors were collected and analyzed using flow cytometry to determine $CD4^+Foxp3^+$ T cells (Treg). ns: not significant.
